# Wire Arc Additive Manufacturing of Aluminum Foams Using TiH_2_-Laced Welding Wires

**DOI:** 10.3390/ma17133176

**Published:** 2024-06-28

**Authors:** Marcel Köhler, Alexander Nikitin, Peter Sonnenfeld, Ralf Ossenbrink, Sven Jüttner

**Affiliations:** 1 Institute of Materials and Joining Technology, Otto-von-Guericke-University Magdeburg, 39106 Magdeburg, Germany; 2 Department of Joining and Welding Technology, Brandenburg University of Technology of Cottbus-Senftenberg, 03046 Cottbus, Germany

**Keywords:** MIG welding, wire arc additive manufacturing, direct energy deposition, aluminum foam, titanium hydride, foaming agent, metal-cored wires, continuous powder extrusion, energy absorption

## Abstract

Composite materials made from aluminum foam are increasingly used in aerospace and automotive industries due to their low density, high energy absorption capacity, and corrosion resistance. Additive manufacturing processes offer several advantages over conventional manufacturing methods, such as the ability to produce significantly more geometrically complex components without the need for expensive tooling. Direct Energy Deposition processes like Wire Arc Additive Manufacturing (WAAM) enable the additive production of near-net-shape components at high build rates. This paper presents a technology for producing aluminum foam structures using WAAM. This paper’s focus is on the development of welding wires that are mixed with a foaming agent (TiH_2_) and produce a foamed weld metal as well as their processing using MIG welding technology.

## 1. Introduction

Aluminum foams are cellular metallic materials characterized by a structure of pores and cell walls made of aluminum. Structurally they can be roughly classified into open-cell and closed-cell foam [1] Open-cell foams have such a high porosity that the pores are not separated from each other, resulting in a continuously open cell structure. Such foams are characterized by a very high specific surface area and high thermal conductivity, and can also be permeated by fluids. Therefore, this type of foam is interesting for thermodynamic or chemical applications (heat exchangers or catalysts) [2].

**Figure 1:**
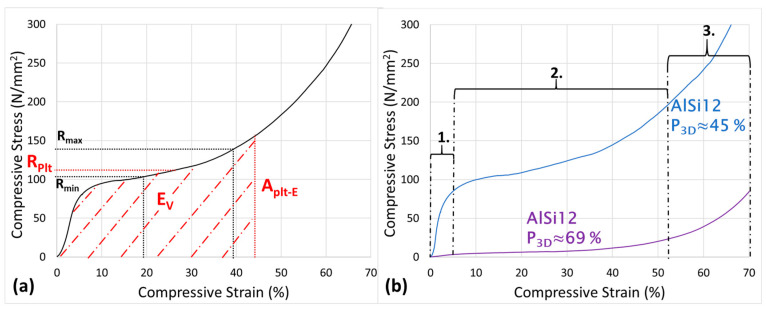
(**a**) Example of a characteristic stress–strain curve for metal foams and corresponding parameters according to DIN 50134:2008-10 [6]; (**b**) example of the mechanical behavior of aluminum foams with different porosities and under quasi-static compressive loading. The numbers 1–3 refer to the 3 stages of deformation described on page 1.

For use as structural materials, open-cell foams are less suitable due to their generally thin cell walls/struts, which result in lower specific strengths. Here, closed-cell aluminum foams (CCAFs) are predominant. These consist of a continuous structure of gas-filled pores surrounded by solid aluminum cell walls. Their mechanical behavior under quasi-static compressive loading can be divided into three phases [3,4,5] (see also Figure 1b):

1. Elastic deformation, where compression and compressive stress are proportional to each other.
2. Formation of a stress plateau, where the compressive stress remains nearly constant (ideally).
3. Compression range, where the compressive stress increases sharply again.

The transitions between these phases can be gradual depending on the material structure, especially for foams with ductile behavior. It is also possible that a foam does not form a plateau in Phase 2 but instead exhibits a continuous (although weaker) stress increase (see Figure 1a). Some foams even show a “brittle” fracture behavior, where individual struts suddenly break, resulting in short drops in compressive stress in the stress–strain diagram. The material behavior and the associated phases are exemplified in stress–strain diagrams shown in (see Figure 1b).

The formation of the stress plateau at relatively low compressive stress results leads to a high energy absorption capacity under compressive loads (e.g., in a crash scenario). The standard DIN 50134:2008-10 describes a quasi-static compression test for metallic foams with a porosity of 50% or higher and serves to determine mechanical properties [6]. In this publication, the focus is primarily on the plateau stress *R_Plt_* [MPa], the strain at the end of the plateau *A_Plt-E_* [-], and the specific energy absorption *E_V_* [MJ/m^3^], which are further explained (see values in Figure 1a).

The plateau stress *R_Plt_* is the compressive stress value at which the stress plateau forms and describes the stress range where the material can effectively absorb energy. For foams with a continuous stress increase, *R_Plt_* is calculated according to DIN 50134:2008-10 as follows:

(1) $$R_{plt}=R_{max}-\frac{\Delta R}{2}=R_{max}-\frac{R_{max}-R_{min}}{2}$$

Here, *R_max_* and *R_min_* are the compressive stress values at strains *ε_max_* = 40% and *ε_min_* = 20%, respectively. The specific energy absorption *E_V_* describes the amount of energy absorbed per foam volume and can be calculated as the area under the stress–strain curve:

(2) $$E_{V}=\int_{e=0}^{A_{Plt-E}}R\left(e\right)\;de_{p}$$

The strain at the end of the plateau *A_Plt-E_* serves as the upper integration limit, which must be calculated in advance using Formula (3):

(3) $$A_{Plt-E}=f(1,3\times R_{Plt})$$

Since the first appearance of aluminum foams in the 1940s, various manufacturing methods for aluminum foams and resulting composite materials have been explored [7]. Excluding additive manufacturing methods these can roughly be divided into two categories: melt metallurgical and powder metallurgical production routes. For the technology presented in this paper, the melt metallurgical production with the addition of blowing agents serves as the basis (also referred to as melt foaming).

**Figure 2:**
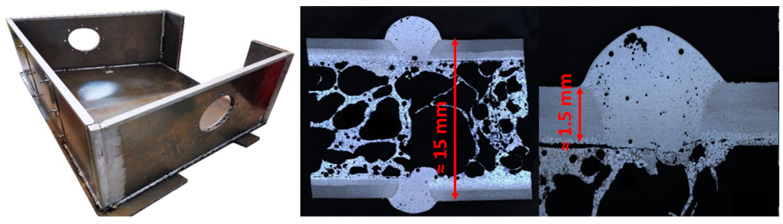
Example of a traction battery case made from aluminum foam sandwiches and corresponding cross-section of MIG welded (manufactured by University of Magdeburg).

In this method, an aluminum alloy is initially melted, thickened by adding metal powders, foamed by adding a blowing agent, and then cooled to solidify [8] (p. 14). Commonly used blowing agents include metal hydrides (TiH_2_, ZrH_2_—releasing H_2_) or carbonates (CaCO_3_, dolomite—releasing CO_2_). For increasing the viscosity of the molten aluminum, substances such as Ca or Al_2_O_3_ are used [4]. Using melt metallurgical methods, it is also possible to produce molded parts from Al foam (analogous to standard casting processes), but this is only economically viable for large-scale production. Aluminum foams are particularly prevalent as sandwich composites with solid aluminum or steel cover sheets. Here, the so-called powder metallurgical route is employed. A powder mixture of Al alloy, blowing agent, and pore-stabilizing agents (e.g., Al_2_O_3_) is initially pressed into a foamable semi-finished product and placed between the cover sheets. Subsequent heat treatment causes the semi-finished products to foam and simultaneously form a metallic bond with the cover sheets [9]. With this technology primarily two-dimensional components (sheets) or profile structures can be produced cost-effectively. These sandwich panels can then be processed, e.g., via MIG welding (for example, see Figure 2). 

The production of metal foams can also be realized using additive manufacturing technologies. Advantages over conventional methods include immense freedom in component geometry without the need for expensive casting tools. Therefore, additive manufacturing holds great potential for small-batch production and the production of structurally optimized components [10]. From the literature, additive methods from the category “Direct Energy Deposition” are known for additive foam production. Specifically, for aluminum foams, laser-based additive processes such as laser powder deposition welding [11,12] or selective laser melting in a powder bed [13] are described in the literature. The production of porous steel using Wire Arc Additive Manufacturing is also known [14] (summary see Table 1).

Table 1 shows that using laser-based additive manufacturing methods for metal foams requires significantly more foaming agent than conventional manufacturing methods (around 1 wt.% for melt foaming [16]) while achieving less porosity (70–80% for melt foaming [16]). Compared to the laser-based additive processes described above, the Wire Arc Additive Manufacturing (WAAM) process investigated here has two distinct advantages:

1. Wire-based additive processes can achieve significantly higher deposition rates, benefiting productivity and cost-effectiveness.
2. The technological basis for WAAM is Metal Inert Gas Welding (MIG). This means that no powder bed is required, and additive structures can be printed on any weldable aluminum substrate (including already existing components).

The main drawbacks of WAAM compared to laser-based methods like SLM are the lack of geometric accuracy and the high energy input when building up parts. In most cases a subsequent cutting process is needed for all functional surfaces. Usually, pores are classified as a defect when welding and creating parts from aluminum via WAAM because they reduce the cross-sectional area of the part, and thus its resistance against tensile loads [17]. However, if the porosity is sufficiently high, advantageous material behavior like high energy absorption capacity under compressive loads can be achieved. 

The WAAM process is implemented analogous to the most well-known additive manufacturing method, Fused Deposition Modeling (FDM), where a plastic filament is continuously fed and melted by a heating coil. WAAM uses a metallic wire electrode that is continuously fed and melted by an electric arc. An important parameter for the energetic description of a welding process is the so-called energy input per unit length *E_L_* [kJ/cm]. It is calculated using Formula (4) [18].

(4) $$E_{L}=\frac{U_{W}\times I_{W}}{v_{w}}$$

Here, *U_W_* and *I_W_* are the average welding voltage and welding current during the welding process, respectively, and *v_W_* is the welding speed, specifically the movement speed of the welding torch.

To realize an additive MIG welding process for aluminum foams, this paper investigates the manufacturing and welding processing of aluminum wire electrodes laced with the blowing agent TiH_2_. Two manufacturing routes for such welding wires are described and compared: the production of metal-cored wire electrodes and the production of solid wire electrodes using a continuous powder extrusion process [19]. The main contributions of this research are: a novel additive processing method for aluminum foam with the potential for high build up rates, and thus high productivity; corresponding material and process requirements; and a comparison between conventionally and additively manufactured aluminum foams regarding their mechanical properties. 

## 2. Materials and Methods

### 2.1. Manufacture of Metal-Cored Welding Wires (Electrodes 1 and 2)

In this study, two specially designed metal-cored wires were investigated. The first cored wire (electrode 1) was developed at the Department of Joining and Welding Technology at BTU Cottbus-Senftenberg. An AlMg1 strip was used as the sheath of this metal-cored wire. The mechanical properties were set according to the DIN EN 485-2 standard [20]. The strip was 10 mm wide and 0.5 mm thick. The powder used as the filling material was a spherical AlSi10Mg powder (45–90 μm) with 10% TiH_2_ powder (5–65 μm). 

The cumulative particle size distribution was assessed using dynamic image analysis with a Camsizer X2 (Microtrac Retsch GmbH, Haan, Germany). The determined particle size distribution parameters were as follows: D10 was measured at 36 µm, D50 at 58 µm, and D90 at 95 µm. The bulk density of the mixed powder was measured using the method according to DIN ISO 3923-2 [21] with a Scott volumeter and was 1.42 g/cm^3^. The flow rate of the mixed powder was determined using the test method according to DIN ISO 4490 [22] with a Hall Flowmeter and was 69 s/50 g.

The precursor wire was produced on a machine from the manufacturer Tianjin Xuzhi (Tianjin, China). For this purpose, the AlMg1 strip was initially formed into a U-shape in two steps and then filled with powder. Subsequently, the strip was closed by roll forming with an overlap and rolled to a diameter of 3.0 mm through 5 pairs of vertical and horizontal rollers for powder compression. Before the precursor wire was drawn to its final diameter of 1.6 mm, a heat treatment was carried out in an air-circulating oven from Nabertherm (New Castle, DE, USA) to remove cold work hardening.

**Figure 3:**
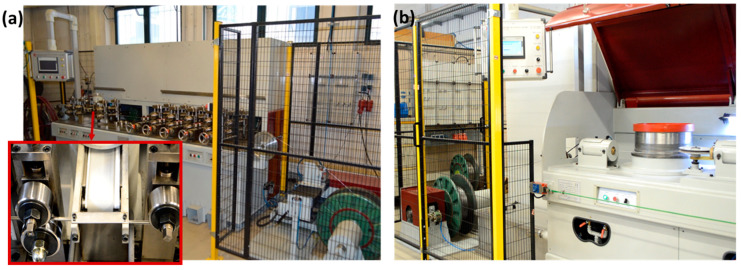
(**a**) Metal-cored wire machines at the BTU Cottbus-Senftenberg; (**b**) wire drawing machine at the BTU Cottbus-Senftenberg.

The drawing of the cored wire was performed on a wire drawing machine from the same manufacturer, Tianjin Xuzhi (Tianjin, China), with 4 consecutive stages. The wire drawing speed was 0.3 m/s. For drawing to the final diameter of 1.6 mm, 3 passes with a total of 12 forming stages were required. The diameter reduction in the drawing stages was up to 12%. Both machines (for metal-cored wire production and for wire drawing) are depicted in Figure 3.

The second metal-cored wire (electrode 2) was manufactured by CORODUR VERSCHLEISS-SCHUTZ GmbH (Thale, Germany). Detailed information about the manufacturing process is not available. Electrode 2 differs from electrode 1 in the sheath material, the composition of the powder filling, and the mass ratio of the powder filling to the total mass of the wire (so-called filling degree). The detailed specifications of the wire composition are summarized in Table 2.

### 2.2. Manufacture of Welding Wires via Continuous Powder Extrusion Process (Electrodes 3 and 4)

**Figure 4:**
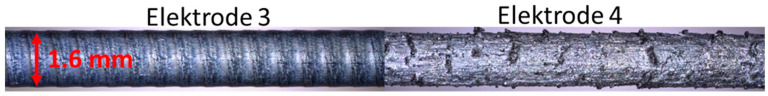
Example of TiH_2_-laced aluminum welding wires (D 1.6 mm) with irregular surfaces.

Electrodes 3 and 4 were produced using a continuous powder extrusion process (trade name TEMCONEX^®^ by Neue Materialien Fürth GmbH, Furth, Germany) through the Leichtmetallkompetenzzentrum Ranshofen GmbH (Braunau am Inn, Austria). With this method, metal in powder form can be continuously processed into a solid profile. The process is used, among other things, to produce foamable semi-finished products for the manufacture of aluminum foam sandwich panels. Therefore, the production of foamable aluminum welding wire electrodes is closely related to this method. For the production of the electrodes, powder mixtures of Al, Si, and Mg, and a defined proportion of the blowing agent TiH_2_, were prepared and pressed into wires with a diameter of 1.6 mm and a length of approximately 5 m. Depending on the size of the powder, an irregular surface structure was created, which must be taken into account during the welding processing (see Figure 4).

**Figure 5:**
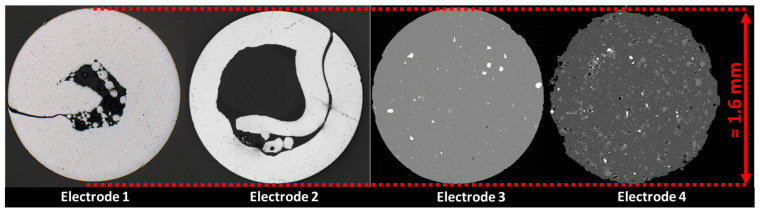
Cross-sections of investigated welding wires (1 and 2: metal-cored wires; 3 and 4: extruded welding wires). Diameter, 1.6 mm.

In Figure 5 cross-sections of the 4 electrodes compared in this publication are depicted. The composition of the electrodes is summarized in Table 2. Due to the preparation, the powder from the cored wire electrodes has partially leaked out and is no longer fully visible in the cross-section.

The material selection was influenced by many factors such as availability and processability of raw materials. The main goal, however, was the production of foams made from unalloyed aluminum (electrode 2), AlSi-based alloys (electrode 4), and AlMgSi-based alloys (electrodes 1 and 3), according to DIN EN 573 [23]. The materials were selected accordingly.

### 2.3. Weld Processing of TiH_2_-Laced Welding Wired

**Figure 6:**
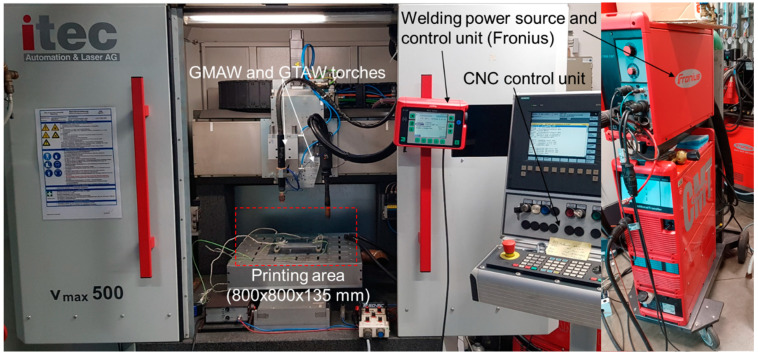
Welding system used to process developed welding electrodes.

The wire electrodes depicted in Figure 5 and Table 2 were processed using a Metal Inert Gas Welding (MIG) process. The equipment used is shown in Figure 6. The figure displays the CNC-controlled test setup equipped with a MIG power source (Fronius CMT Advanced 4000 (Fronius International GmbH, Wels, Austria)). The welding power source was thereby used for process control and the recording of the welding voltage/welding current.

For processing metal-cored wires, a highly energy reduced welding process is required. Therefore, an energy reduced short arc and reverse wire feed was chosen as the process (Trade name “Cold Metal Transfer”, positive polarity on the wire electrode). Table 3 summarizes the corresponding process parameters of the welding experiments.

For the welding processing of the cored wires, particularly the 4 wire feed rollers in the wire feed unit and in the torch feed had to be adjusted. To transfer the feed motion to the cored wires, thicker feed rollers (designed for 1.2 mm diameter wire) were used for a wire diameter of 1.6 mm. Similarly, the pressure roller in the torch was replaced. When processing some extruded electrodes (electrode 4), the surface of the electrodes had to be manually ground down since the rough surface led to feeding issues. The 5 m wire segments had to be inserted from the front into the torch neck and fed backwards into the hose package before processing.

### 2.4. Methods of Analysis

**Figure 7:**
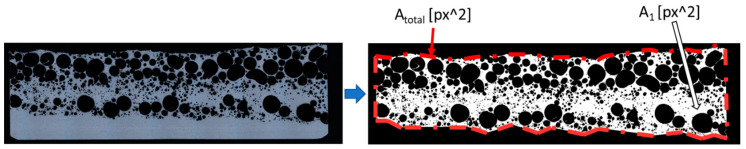
Determination of porosity in longitudinal cross-sections of single bead deposition welds (A_total_ refers to the total area of the weld outlined by the red arrow/frame, A_1_ refers to white area (solid aluminum without pores)).

Porosity is one of the key parameters for characterizing a foam structure. Depending on the sample, two methods were applied for porosity determination. With the electrodes shown in Table 2, single-layer weld overlays were first welded onto an AlMg3 sheet substrate to determine the porosity *P*_2*D*_ in the weld metal based on cross-sectional images (plane of the image parallel to the welding direction). Samples of 30 mm in length were taken from the approximately 100 mm long weld beads at the midpoint. To quantify the porosity in cross-sectional images (in 2D), the open-source image processing software ImageJ2 1.53q with the image processing package Fiji were used (available at [24]). As shown in Figure 7 and Equation (5), ImageJ can be used to calculate an area ratio between the total area of the weld *A_total_* and the area of solid aluminum *A*_1_.

(5) $$P_{2D}=100\times \left(1-\frac{A_{1}}{A_{total}}\right)$$

To determine the porosity in block structures *P*_3*D*_, the sample volume *V_sample_* [cm^3^] was first calculated by measuring the edge lengths, and the sample mass *m_sample_* [g] was measured using a precision scale. With this information, the sample density *ρ_sample_* [g/cm^3^] was calculated. Using the density of the alloy *ρ_alloy_* [g/cm^3^], which can be obtained from material data sheets (Supplementary Table S1), the porosity was calculated using the following formula:

(6) $$P_{3D}=100\times \left(1-\frac{\rho_{sample}}{\rho_{alloy}}\right)$$

**Figure 8:**
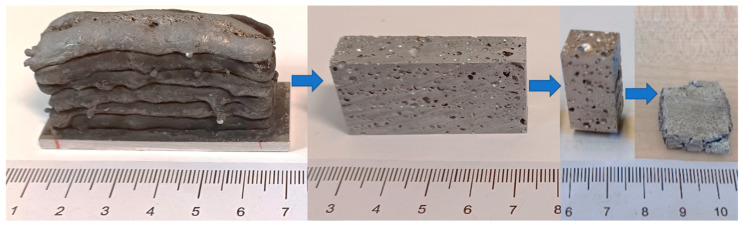
Example of additively manufactured aluminum foam structures after welding, mechanical processing, and compression testing (electrode 3, P_3D_ ≈ 41%).

The mechanical properties of the foams were determined according to DIN 50134, as described. Initially, the average pore diameter was calculated using the ImageJ 1.53q software based on cross-sectional images. The foam blocks were then cut into test specimens with standardized dimensions using a wet cutting grinding machine and compressed up to 80% at a compression rate of ė = 5 × 10^−3^ s^−1^ using a universal testing machine. The sample preparation process is shown in Figure 8).

The welding process was visualized using a high-speed camera (Photron FASTCAM SA3 with a 12× zoom lens from NAVITAR, Rochester, NY, USA) at a frame rate of 3000 frames per second. To visualize the structure of the foam blocks in three dimensions, one block was analyzed for porosity using computed tomography (“phoenix nanotom m” from Waygate Technologies, Hürth, Germany) as an example.

## 3. Results and Discussion

### 3.1. Porosity in Single Deposition Weld Beads

**Figure 9:**
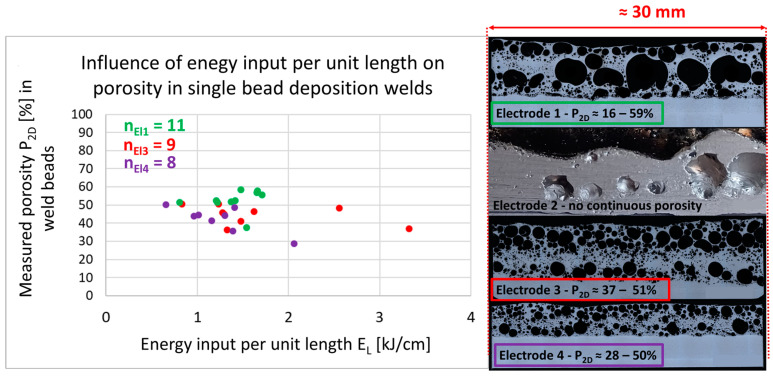
Longitudinal cross-sections of high porosity deposition weld beads with corresponding porosity value–examples and influence of energy input per unit length *E_L_*.

During the processing of the cored wires, welding processes were realized with an energy input range of approximately 0.8–3.2 kJ/cm. Within this range no significant influence of the energy input per unit length on the achieved porosity could reliably be determined (see Figure 9).

**Figure 10:**
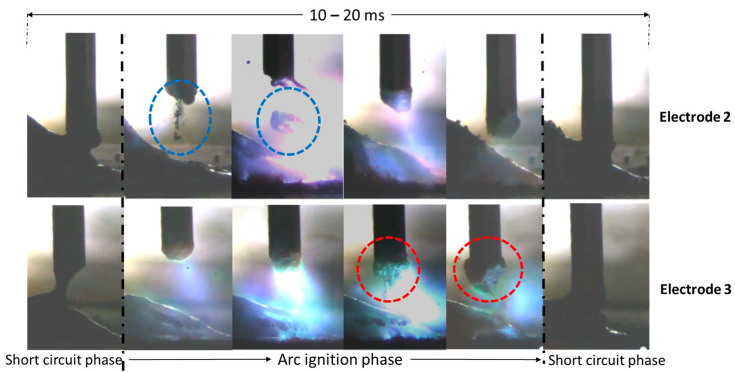
Excerpt of high-speed-video of droplet formation using an energy reduced short arc.

It is noticeable that three out of the four processed electrodes delivered similar porosity results, even though electrode 1 had a significantly higher blowing agent content (3.3%) compared to electrodes 3 and 4 (each 0.8%). One possible explanation is the observed powder loss during the welding of the metal-cored wire electrodes. With electrode 2, although porous welds could be produced, the pore distribution and the seam geometry were so irregular that a more detailed evaluation was waived. Electrode 4 showed a slight decrease in porosity when increasing *E_L_.* However, due to high process fluctuations and a small sample size, no correlation should be assumed here. Recordings with a high-speed camera showed that the energy reduced short arc process could not be reliably implemented with electrode 2. In the arc ignition phase, the sheath of the cored wire partially melted away, abruptly exposing the powder before the directed droplet transfer could occur during the short circuit (see blue circles in Figure 10). This effect was not observed with the extruded electrodes. Here, it was observed instead that the droplet “boils” during the arc phase and then transitions directionally into the molten pool (see red circles in Figure 10). 

From a welding perspective, electrode 3 was the most easily processed. With this electrode, an energy-reduced short arc process could be realized with minimal spattering, resulting in a uniform, highly porous weld seam with a closed surface.

### 3.2. Porosity and Mechanical Properties of Three-Dimensional Block Structures 

**Figure 11:**
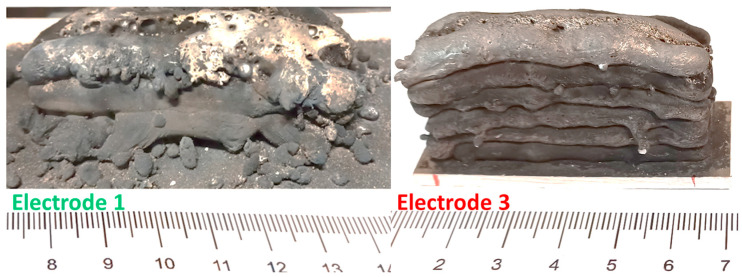
Results from additive welding experiments with TiH_2_-laced welding wires.

With electrodes 1, 3, and 4, additive block structures made of aluminum foam were produced and their behavior under compressive load was tested according to DIN 50134:2008-10. Similar to the previously described experiments no significant and uniform porosity could be achieved with electrode 2. It was also observed that only the electrodes produced by powder extrusion (3 and 4) were well-suited for multi-layer welding. Electrode 1 (metal-cored wire) tended to produce excessive spattering, powder loss, and deposition of oxides on the weld seam surface during multi-layer build-up (see Figure 11). However, this effect could likely be counteracted by optimizing the characteristic curve and the welding parameters.

**Figure 12:**
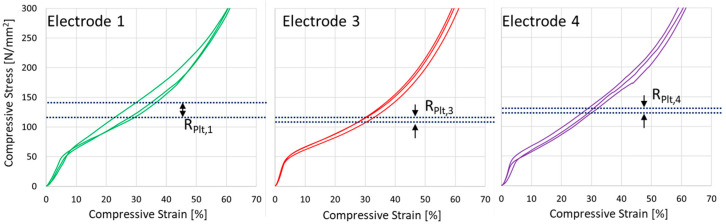
Mechanical behavior of additively manufactured aluminum foams under uniaxial compression (according to DIN 50134:2008-10) and respective plateau stress *R_Plt_* (see also Table 4).

The results are presented in the form of stress–strain diagrams in Figure 12. Additionally, selected mechanical properties are shown in Table 4 and compared with the literature references.

The inverse proportionality between porosity and mechanical properties known from the literature [4] can be seen when comparing the findings from this study with the reference values. In comparison to the reference foams from the literature, the additively manufactured foams described here achieve significantly lower porosity in some cases. However, the mechanical properties such as plateau stress and energy absorption capacity are many times higher. Therefore, the additively manufactured foams could absorb the same amount of impact energy with significantly less material. It should be noted that the additive foams only effectively absorb energy at much higher stresses. A general statement about their effectiveness cannot be made. The porosity range seems to vary between different electrodes. However, due to the small sample size per electrode, no trend can be observed properly. A possible explanation for the high variation in porosity within samples made from electrode 1 are process instabilities and the powder loss described earlier. 

**Figure 13:**
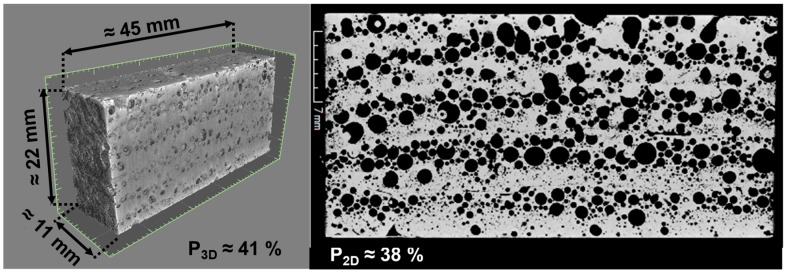
CT-Scan of additively manufactured aluminum foam block (electrode 3) with corresponding porosity in three and two dimensions.

For the interpretation of the properties, a foam block was measured exemplarily using computed tomography and a volumetric body was reconstructed (see Figure 13). The reconstruction shows that while structures with high porosity can be created, some of the pores are destroyed when building up layers above previously welded seams. This explains the continuous stress increase in the compression test. The porous structure and the solid material deform simultaneously. Similar mechanical behavior is also described in [13] for foams manufactured additively using selective laser melting from a powder bed. Here, the relatively low porosity is cited as the main reason for the continuous stress increase.

## 4. Conclusions

The goal was to produce aluminum foams using additive MIG welding technology. For this purpose, four different wire electrodes from two different manufacturing routes were produced, processed via MIG welding, and compared in terms of their weldability and foam properties. The comparison is summarized in Table 5.

In the end, only electrode 3 delivered good welding results for single- and multi-layer welding. From a technical perspective, wire electrodes extruded from powder are significantly better suited than Al-filled wires for the additive manufacturing of Al foams (stable welding process, less blowing agent usage). However, processing the extruded wire electrodes is only trouble-free if sufficiently fine powder is used for manufacturing (ideally < 150 µm). Otherwise, the irregular wire surface causes conveying problems (limited suitability of electrode 4). For the filled wire electrodes, a significant optimization effort regarding composition and welding process would be necessary for comparable results. To further improve the foam structure, it is necessary to minimize the proportion of destroyed structure during re-welding. For maintaining the structure, additional approaches should be considered in the future, such as the use of hydrogen-containing shielding gases.

## Figures and Tables

**Table 1:** Comparison of various publications on the additive manufacturing of metal foams.

Author	Ref.	Manufacturing Process	Alloy	Gas Injection Via	Porosity [%]
Feng et al.	[11]	Laser powder deposition welding	AlSi10Mg	Ni-coated TiH_2_ (4–11.5 wt.%)	36–70
Shim et al.	[12]	Laser powder deposition welding	AlSi12	ZrH_2_ (10–40 wt.%)	0.1–24.8
Zhang et al.	[13]	Selective laser melting	AlSi12	TiH_2_ (5–10 wt.%)	22–38
Ren et al.	[14]	Wire Arc Additive Manufacturing	Er50-6 (low carbon steel)	shielding gas (80% Ar, 20% CO_2_) + air	64–87
An et al.	[15]	Selective laser melting	AlSi12	CaCO_3_ (5–15 wt.%)	26.9–39.6

**Table 2:** Overview of investigated electrodes; metal-cored wires—No. 1 and 2; and wires manufactured via continuous powder extrusion—No. 3 und 4 (manufacturer’s data).

**No.**	**Strip Material**	**Powder Filling (wt.%)**	**Filling Degree [wt.%]**	**Amount of TiH_2_ [wt.%]**	**Type of Metal-Cored Wire**
1	AlMg1 (10 × 0.5 mm)	AlSi10Mg + 10% TiH_2_	33	3.3	Seamed/straight limbs
2	Al99.5 (8 × 0.29 mm)	Al + 16% TiH_2_ + 33.8% Al_2_O_2_	27	4.3	Seamed/overlapping
**No.**	**Composition of powder mixture [wt.%]**		**Particle size [** **µm]**	**Amount of TiH_2_ [wt.%]**	**Process/type**
3	AlMg1Si0.6 + 0.8 TiH_2_		+63/−150	0.8	Solid wire Electrode/ continuously extruded
4	AlSi10 + 0.8% TiH_2_		<500	0.8	

**Table 3:** Overview of functioning welding parameters.

Electrode	Substrate	Geometries	Contact Tube Distance [mm]	Shielding Gas	Position	v_s_ [cm/min]	v_D_ [m/min]	E_W_ [kJ/cm]
1	AlMg3, 150 × 150 × 3 mm	Single beads; block structures	18	Ar 4.7 (15–17 L/min)	PA	600–1000	4–8	0.80–1.37
2						500–1000	5–7	0.94–2.42
3						500–1000	6–8	1.24–3.32
4						600–1000	5–6	0.90–1.82

**Table 4:** Results of uniaxial compression testing according to DIN 50134:2008-10 with reference values taken from [3] (p. 10).

**Electrode**	**Number of samples**	**ρ [g/cm^3^]**	**ρ_rel_ [%]**	***P*_3*D*_ [%]**	***R_Plt_* [N/mm^2^]**	***E_V_* [MJ/m^3^]**
1	3	1.51–1.89	56–70	30–44	124–143	37.8–43.4
3	4	≈1.58	≈59	≈41	113–122	34.3–37.1
4	3	1.53–1.59	57–59	41–43	124–137	35.3–40.4
Reference values taken from [3] (p. 10) with respective manufacturing method						
**Manufacturing method**		**ρ [g/cm^3^]**	**ρ_rel_ [%]**	***P* [%]**	***R_Plt_* [N/mm^2^]**	***E_V_* [MJ/m^3^]**
Modified alporas route		-	13.4–47.2	52.8–86.6	2.8–23.5	1.7–18.9
As recieved Alulight		-	10–50	50–90	3.3–12.7	-
Dynamic gas injection		-	29.1	70.9	21.6	11.1
Closed-cell aluminum composite foams (reinforced by CNTs) manufactured via friction stir welding		-	54.2–69.8	30.2–45.8	-	6.63–11.45

**Table 5:** Summary of suitability of TiH_2_-laced aluminum welding wires for additive foam manufacturing.

Electrode No.	1	2	3	4
Welding process realizable (energy rediced short arc)	Yes	Limited	Yes	Limited
Suitability for single layer deposition welding	Yes	No	Yes	Limited
Porosity in single weld bead (*P*_2*D*_ [%])	16–59	-	37–51	28–50
Suitability for WAAM process with multiple layers	Limited	No	Yes	No
Porosity in block structure (*P*_3*D*_ [%])	30–44	-	≈41	41–43

## Data Availability

The raw data supporting the conclusions of this article will be made available by the authors on request.

## Supplementary Materials

The following supporting information can be downloaded at: https://www.mdpi.com/article/10.3390/ma17133176/s1, Supplementary Table S1: Welding Parameters and Porosity Measurement in Single Bead Deposition Welds.

## References

[1] Pandey R., Singh P., Khanna M., Manufacturing Q.M.M.F. Mechanical Properties and Its Designing Aspects—A Review. Proceedings of the ICAPIE 2019: Advances in Manufacturing and Industrial Engineering.

[2] Wan T., Liu Y., Chen X., Li Y. (2021). Fabrication, properties, and applications of open-cell aluminum foams: A review. J. Mater. Sci. Technol..

[3] Nisa S.U., Pandey S., Pandey P.M. (2022). A review of the compressive properties of closed-cell aluminum metal foams. J. Process Mech. Eng..

[4] Byakova A., Gnyloskurenko S., Vlasov A., Yevych Y., Semenov N. (2022). The Mechanical Performance of Aluminum Foam Fabricated by Melt Processing with Different Foaming Agents: A Comparative Analysis. Metals.

[5] Yu C.-J., Banhart J. (1998). Mechanical Properties of Metal Faoms. Bremen. https://www.researchgate.net/publication/283363376_Mechanical_properties_of_metal_foams/.

[6] (2008). Prüfung von Metallischen Werkstoffen—Druckversuch an Metallischen Zellularen Werkstoffen.

[7] Ji C., Huang H., Wang T., Huang Q. (2023). Recent advances and future trends in processing methods and characterization technologies of aluminum foam composite structures: A review. J. Manuf. Process..

[8] Parveez B., Jamal N.A., Anuar H., Ahmad A., Aabid A., Baig M. (2022). Microstructure and Mechanical Properties of Metal Foams Fabricated via Melt Foaming and Powder Metallurgy Technique: A Review. Materials.

[9] Hommel P., Roth D., Binz H. Deficits in the Application of Aluminum Foam Sandwich: An Industrial Perspective. Proceedings of the Design Society: Design Conference.

[10] Treutler K., Wesling V. (2021). The Current State of Research of Wire Arc Additive Manufacturing (WAAM): A Review. Appl. Sci..

[11] Feng X., Zhang Z., Cui X., Jin G., Zheng W., Liu H. (2018). Additive manufactured closed-cell aluminum alloy foams via laser melting deposition process. Mater. Lett..

[12] Shim D.-S. (2021). Effects of process parameters on additive manufacturing of aluminum porous materials and their optimization using response surface method. J. Mater. Res. Technol..

[13] Zhang M., Chen C., Huang Y. (2017). Laser additive manufacturing foam aluminium-12 wt-% silicon with different addition TiH2 foaming agent. Mater. Sci. Technol..

[14] Ren D., Ba X., Zhang Z., Zhang Z., Zhao K., Liu L. (2023). Wire arc additive manufacturing of porous metal using welding pore defects. Mater. Des..

[15] An J., Chen C., Zhang M. (2021). Effect of CaCO3 content change on the production of closed-cell aluminum foam by selective laser melting. Opt. Laser Technol..

[16] Byakova A., Gnyloskurenko S., Sirko A. (2006). The Role of Foaming Agent in Structure and Mechanical Performance of Al Based Foams. Mater. Trans. Spec. Issue Porous Foam. Met.—Fabr. Charact. Prop. Appl..

[17] Hauser T., Reisch R.T., Kaplan A.F.H. (2021). Porosity in wire arc additive manufacturing of aluminium alloys. Addit. Manuf..

[18] (2015). Welding and Allied Processes–Guidelines for Measurement of Welding Energies.

[19] Neue Materialien Fürth GmbH Temconex (R)-Technologie. September 2018. https://nmfgmbh.de/wp-content/uploads/2018/08/180814_Temconex_deutsch.pdf.

[20] (2018). Aluminium and Aluminium Alloys—Sheet, Strip and Plate—Part 2: Mechanical Properties.

[21] (1987). Metallic Powders—Determination of Apparten Denisty; Scott Volumeter Method; Identical to ISO 3923/2.

[22] (2018). Metallic Powders—Determination of Flow Rate by Means of a Calibrated Funne (Hall Flowmeter).

[23] (2024). Aluminium and Aluminium Alloys—Chemical Composition and Form of Wrought Products—Part 3: Chemical Composition and Form of Products.

[24] Binz H., Honold C., Bauernhansl T., Konstruktion M.S. Herstellung eines Auslegers aus Aluminiumschaum-Sandwich unter Anwendung der Fräskanttechnik. VDI Fachmedien GmbH & Co. KG Unternehmen für Fachinformationen, 1 August 2018. https://www.ingenieur.de/fachmedien/konstruktion/werkstoffe/konstruktion-und-herstellung-eines-auslegers-aus-aluminiumschaum-sandwich-unter-anwendung-der-fraeskanttechnik/.

